# Development of a Machine Learning-Based Autophagy-Related lncRNA Signature to Improve Prognosis Prediction in Osteosarcoma Patients

**DOI:** 10.3389/fmolb.2021.615084

**Published:** 2021-05-21

**Authors:** Guang-Zhi Zhang, Zuo-Long Wu, Chun-Ying Li, En-Hui Ren, Wen-Hua Yuan, Ya-Jun Deng, Qi-Qi Xie

**Affiliations:** ^1^The Second Clinical Medical College, Lanzhou University, Lanzhou, China; ^2^Department of Orthopedics, Lanzhou University Second Hospital, Lanzhou, China; ^3^Lintao County Traditional Chinese Medicine Hospital of Gansu Province, Lintao, China; ^4^The Fourth People’s Hospital of Qinghai Province, Xining, China; ^5^Department of Orthopaedics, Xining First People’s Hospital, Xining, China; ^6^Affiliated Hospital of Qinghai University, Xining, China; ^7^Affiliated Cancer Hospital of Qinghai University, Xining, China; ^8^Breast Disease Diagnosis and Treatment Center, Affiliated Hospital of Qinghai University & Affiliated Cancer Hospital of Qinghai University, Xining, China

**Keywords:** osteosarcoma, autophagy-related lncRNA, prognostic signature, survival, immune cell infiltration

## Abstract

**Background:**

Osteosarcoma is a frequent bone malignancy in children and young adults. Despite the availability of some prognostic biomarkers, most of them fail to accurately predict prognosis in osteosarcoma patients. In this study, we used bioinformatics tools and machine learning algorithms to establish an autophagy-related long non-coding RNA (lncRNA) signature to predict the prognosis of osteosarcoma patients.

**Methods:**

We obtained expression and clinical data from osteosarcoma patients in the Therapeutically Applicable Research to Generate Effective Treatments (TARGET) and Gene Expression Omnibus (GEO) databases. We acquired an autophagy gene list from the Human Autophagy Database (HADb) and identified autophagy-related lncRNAs by co-expression analyses. Gene Ontology (GO) and Kyoto Encyclopedia of Genes and Genomes (KEGG) pathway enrichment analyses of the autophagy-related lncRNAs were conducted. Univariate and multivariate Cox regression analyses were performed to assess the prognostic value of the autophagy-related lncRNA signature and validate the relationship between the signature and osteosarcoma patient survival in an independent cohort. We also investigated the relationship between the signature and immune cell infiltration.

**Results:**

We initially identified 69 autophagy-related lncRNAs, 13 of which were significant predictors of overall survival in osteosarcoma patients. Kaplan-Meier analyses revealed that the 13 autophagy-related lncRNAs could stratify patients based on their outcomes. Receiver operating characteristic curve analyses confirmed the superior prognostic value of the lncRNA signature compared to clinically used prognostic biomarkers. Importantly, the autophagy-related lncRNA signature predicted patient prognosis independently of clinicopathological characteristics. Furthermore, we found that the expression levels of the autophagy-related lncRNA signature were significantly associated with the infiltration levels of different immune cell subsets, including T cells, NK cells, and dendritic cells.

**Conclusion:**

The autophagy-related lncRNA signature established here is an independent and robust predictor of osteosarcoma patient survival. Our findings also suggest that the expression of these 13 autophagy-related lncRNAs may promote osteosarcoma progression by regulating immune cell infiltration in the tumor microenvironment.

## Introduction

Osteosarcoma is a common bone malignancy in children and young adults (Wedekind et al., 2018; Heymann et al., 2019). It is characterized by high metastasis and recurrence rates, with approximately 10–20% of patients with metastatic osteosarcoma experiencing pain and swelling (Liu et al., 2018). Osteosarcoma patients are typically treated with chemotherapy, radiotherapy, and surgery. Although recent clinical studies have shown that adjuvant chemotherapy can improve the survival rate of such patients, the prognosis remains poor (Tian et al., 2019; Wang J. et al., 2019). Therefore, the development of improved management and risk stratification methods, as well as the identification of robust prognostic biomarkers, are of high clinical importance.

Autophagy is a highly conserved physiological process involving the degradation of damaged proteins and cellular organelles through the formation of autophagosomes and their subsequent fusion with lysosomes (Nadjmi et al., 1987; Ravanan et al., 2017; Levine and Kroemer, 2019; Wong et al., 2020). Mounting evidence indicates that autophagy is a key determinant of cancer progression by regulating cell growth, metastasis, and response to chemotherapy (Folkerts et al., 2019). However, it has also become evident that autophagy is a double-edged sword. In the early stages of cancer, autophagy promotes the degradation of damaged proteins or organelles, thereby preventing cell damage and chromosomal instability and inhibiting cancer progression. On the other hand, once the cancer is formed, autophagy facilitates tumor cell survival under various environmental stresses, such as nutrient deprivation and hypoxia, promoting tumor progression and therapeutic resistance (Levy et al., 2017; Amaravadi et al., 2019; Cuomo et al., 2019). The role of autophagy in osteosarcoma has previously been reported. Notably, in mouse osteosarcoma models, silencing of the autophagy-promoting gene *BECN1* has been shown to enhance cancer cell metastasis (Zhang et al., 2018). Autophagy modulation in the human osteosarcoma cell line MG-63 significantly enhances cell survival and chemoresistance to cisplatin (Kim et al., 2017). Recent studies have shown that autophagy is closely related to immune cell infiltration in the tumor microenvironment (Dehne et al., 2017). Chen D.P. et al. (2018) showed that autophagy induction in liver cancer cells promotes the epithelial–mesenchymal transition (EMT) and metastasis by activating the NF-κB/SNAI1 signaling pathway. Nevertheless, the role of autophagy in osteosarcoma cell metastasis is less clear.

Long non-coding RNA (lncRNA) is a novel family of non-coding RNAs that are more than 200 nucleotides long (Zhao G.S. et al., 2018; Chi et al., 2019). They regulate gene expression at the transcriptional and post-transcriptional levels and play a protumorigenic or tumor suppressor role in various human cancers, including breast, gastric, colorectal, and liver cancers (Choi et al., 2019; Feng et al., 2019; Ni et al., 2019; Song et al., 2019). Increasing evidence shows that the role of lncRNAs in cancer pathogenesis is partly mediated by the regulation of various autophagy-related proteins, in addition to proteins involved in cell proliferation, apoptosis, and metastasis (Chi et al., 2019; Jiang N. et al., 2019; Zheng et al., 2019). For instance, the lncRNA XIST inhibits autophagy in osteosarcoma cells by acting as a sponge for miR-375–3p, thereby regulating the AKT/mTOR signaling pathway (Sun et al., 2019). Similarly, the lncRNA Sox2OT-V7 regulates ULK1, ATG4A, and ATG5 through miR-142/miR-22 to modulate autophagy in osteosarcoma cells (Zhu et al., 2020). The lncRNA LINC00313 acts as a sponge for miR-342–3p, regulating FOSL2 levels and inhibiting autophagy in human osteosarcoma cells, ultimately suppressing osteosarcoma cell metastasis (Chen et al., 2020). LncRNA-ATB modulates autophagy in liver cancer cells by activating Yes-associated protein (YAP) and inducing ATG5 expression, associated with poor prognosis of liver cancer patients (Wang C.Z. et al., 2019). By regulating the expression levels of autophagy-related genes, lncRNAs regulate all stages of autophagy, including initiation, vesicle nucleation, autophagosome maturation, and autophagosome fusion (Pan et al., 2018; Tu et al., 2019). Therefore, autophagy-related lncRNAs may have potential prognostic and therapeutic value in osteosarcoma patients.

**FIGURE 1:**
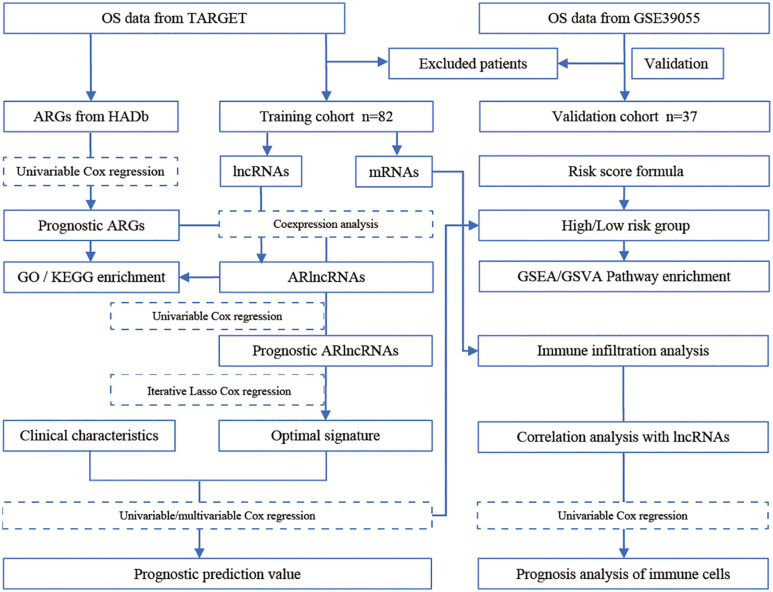
Study flowchart of the identification of a prognostic lncRNA signature in osteosarcoma.

In this study, we used a bioinformatics approach to construct a co-expression network of autophagy-related lncRNA and identify an autophagy-related lncRNA signature based on machine learning algorithms (Figure 1). Furthermore, we analyzed the relationship between autophagy-related lncRNAs and immune cell infiltration in osteosarcoma. Our findings provide further insight into the role of autophagy-related lncRNAs in osteosarcoma and suggest that they are promising prognostic markers and therapeutic targets.

## Materials and Methods

### Data Acquisition, Processing, and Screening

Therapeutically Applicable Research to Generate Effective Treatments (TARGET)^1^ is an open database for childhood cancers. It seeks to use a comprehensive genomic approach to identify molecular changes in the occurrence and development of childhood cancers that are difficult to treat (Gao et al., 2020; Zhang et al., 2020). We obtained gene expression and clinical data of osteosarcoma patients from the TARGET database. After excluding samples lacking survival data, data from the remaining 82 osteosarcoma samples were further analyzed. The GSE39055 dataset was downloaded from the Gene Expression Omnibus (GEO) database (Barrett et al., 2013). It involved data from 37 unique diagnostic biopsy specimens analyzed on the GPL14951 platform (Illumina HumanHT-12 WG-DASL V4.0 R2 expression bead chip) (Kelly et al., 2013). We obtained an autophagy gene list from the Human Autophagy Database (HADb)^2^ and extracted the autophagy-related gene expression matrix using TARGET as the training set and GSE39055 as the validation set. After identifying mRNAs and lncRNAs in the gene expression matrix of the training set, we extracted the lncRNA expression matrix and calculated the Pearson coefficient of lncRNA gene expression and autophagy genes. LncRNAs with Pearson coefficient > ± 0.4 and *P* < 0.05 were considered autophagy-related lncRNAs.

### Co-expression Analyses of Autophagy-Related lncRNAs, Gene Ontology (GO), and Kyoto Encyclopedia of Genes and Genomes (KEGG) Pathway Enrichment Analyses

To assess the potential role of autophagy-related lncRNAs in osteosarcoma development, we performed co-expression analyses using the mRNA and autophagy-related lncRNA expression matrixes. RNAs with Pearson coefficients > ± 0.4 and *P* < 0.05 were considered autophagy-related lncRNA target genes. These genes were subjected to GO and KEGG pathway enrichment analyses using the clusterProfiler package (Yu et al., 2012) and a significance threshold of adjusted *P*-value <0.05.

### Construction and Validation of the Prognostic Model

Iterative lasso regression was used to identify high-frequency features (consensus genes). Consensus genes were incorporated into the Cox model until the area under the curve (AUC) in receiver operating characteristic (ROC) analyses reached a peak, at which point the model was considered optimal and contained the least features (Pungpapong et al., 2020). Next, we performed univariate Cox regression analyses using the obtained autophagy-related lncRNAs to identify autophagy-related lncRNAs associated with osteosarcoma prognosis (*P* < 0.05). Subsequently, we used the glmnet^3^ package to perform machine learning-based lasso Cox regression analyses using autophagy-related lncRNAs to identify the optimal prognostic signature. We identified the consensus genes (frequency > 50) after 500 lasso Cox regressions and performed multivariate Cox regression analyses using the consensus genes. After identifying the optimal prognostic signature, we calculated a prognostic score for each osteosarcoma sample and stratified patients into high and low risk according to the median value of the prognostic score. We used the R package to draw a risk factor chart to show the prognostic score of patients in the high- and low-risk groups. Diferential expressed genes (DEGs) were obtained by analyzing the gene expression matrix using the limma package (Li et al., 2013). The screening criteria were: | log2 fold change (log2FC) | > 1, adjust *P* < 0.05.

### Comparison of the lncRNA Signature With Other Prognostic Biomarkers and Validation in an Independent Cohort

Many prognostic biomarkers have been reported for osteosarcoma. For example, the immune-related gene SP140 is a biomarker of poor prognosis in osteosarcoma (Hong et al., 2020), CCNE1 is a prognostic biomarker and potential therapeutic target (Wei et al., 2020), and SNHG3 is selectively expressed in osteosarcoma tissues (Chen et al., 2019). In addition, miR-221 expression is elevated in osteosarcoma, and its levels are associated with poor prognosis (Yang et al., 2015). The time-dependent receiver operating characteristic (ROC) curve was drawn to evaluate the predictive value of the prognostic gene signature for overall survival using the R package “survivalROC” (Heagerty et al., 2000). To compare the prognostic value of our signature with that of known biomarkers, we conducted comparative time-dependent ROC analyses. We also assessed the prognostic value of our lncRNA signature in an independent cohort (using the GSE39055 dataset).

### Assessment of the lncRNA Signature as an Independent Prognostic Factor and Nomogram Construction

To further assess the potential use of the lncRNA signature as an independent prognostic factor, we performed Kaplan-Meier survival analyses on osteosarcoma patients grouped based on their clinicopathological characteristics. We also performed univariate and multivariate Cox regression analyses. To establish a quantitative method for predicting osteosarcoma patient prognosis, we constructed a nomogram integrating the lncRNA signature and different clinicopathological factors (Iasonos et al., 2008). Then we used a decision curve to compare the prognostic performance of our model with that of the traditional staging system.

### Gene Set Enrichment Analyses (GSEA) and Gene Set Variation Analyses (GSVA) of High- and Low-Risk Osteosarcoma Patients

To compare the gene expression profiles between high- and low-risk osteosarcoma patients, we conducted GSEA and GSVA analyses. For GSEA, we used “c2.all.v7.1.symbols.gm,” “c5.all.v7.1.symbols.gmt,” and “h.all.v7.1.symbols.gmt” as reference gene sets. The analyses were conducted using GSEA 4.0.3 software and significance thresholds of nominal *P* < 0.05 and false discovery rate (FDR) <0.05. For GSVA, we used “h.all.v7.1.symbols.gmt” as the reference gene set. GSVA was conducted using the clusterProfiler and GSVA packages (Hänzelmann et al., 2013) with a significance threshold of adjusted *P*-value <0.05.

### Immune Cell Infiltration Analyses

CIBERSORT uses a linear support vector regression to deconvolve the transcriptome expression matrix and estimate the composition and abundance of immune cells within a mixed cell population (Chen B. et al., 2018). To explore the relationship between the 13 prognostic autophagy-related lncRNAs and immune cell infiltration, we uploaded the gene expression matrix data of osteosarcoma patients to CIBERSORT and filtered for the samples with *P* < 0.05. The obtained matrix was analyzed using the ggplot2 package x to assess the composition of immune cell infiltrate and determine the differences in immune cell infiltration between osteosarcoma tissues and normal tissues. Subsequently, we performed univariate Cox regression analyses to identify immune cells associated with osteosarcoma patient prognosis (*P* < 0.05).

### Correlation Analyses Between Autophagy-Related lncRNAs and Immune Cells

To further analyze the relationship between autophagy-related lncRNAs and immune cells, we performed Spearman correlation analyses using a monotonic equation to evaluate the correlation between the levels of autophagy-related lncRNAs and immune cells.

## Results

### Identification of Autophagy-Related lncRNAs

The gene expression profile data of OS patients and the corresponding clinical data were downloaded from the TARGET database, and excluded samples without survival data. A total of 82 OS samples were included in this study, and the clinicopathological characteristics of whom are summarized in Table 1. The dataset GSE39055 was downloaded from the GEO database, including 37 unique diagnostic biopsy specimens. We download the autophagy gene list from the HADb website, and extracted the autophagy-related gene expression matrix. TARGET was used as the training set, GSE39055 was used as the verification set, and we classified mRNA and lncRNA on the gene list of the expression matrix of the training set and extracted the lncRNA expression matrix. After background correction, data normalization, and removal of inter-batch differences, we identified a total of 222 autophagy-related genes.

**TABLE 1:** Clinicopathological characteristics of osteosarcoma patients.

Characteristics	Groups	Patients (*N* = 82)	
		No.	%
Age	Median	4–32	
	Range	15	
	<18	64	78.05
	≥18	18	21.95
Sex	Male	46	56.10
	Female	36	43.90
Metastatic	Metastatic	20	24.39
	Non-metastatic	62	75.61
Primary tumor site	Arm/hand	6	7.32
	Leg/Foot	74	90.24
	Pelvis	2	2.44
Histological stage	Stage 1/2	24	29.27
	Stage 3/4	18	21.95

### Co-expression Analyses of Autophagy-Related lncRNAs and Enrichment Analyses of GO and KEGG Pathways

**FIGURE 2:**
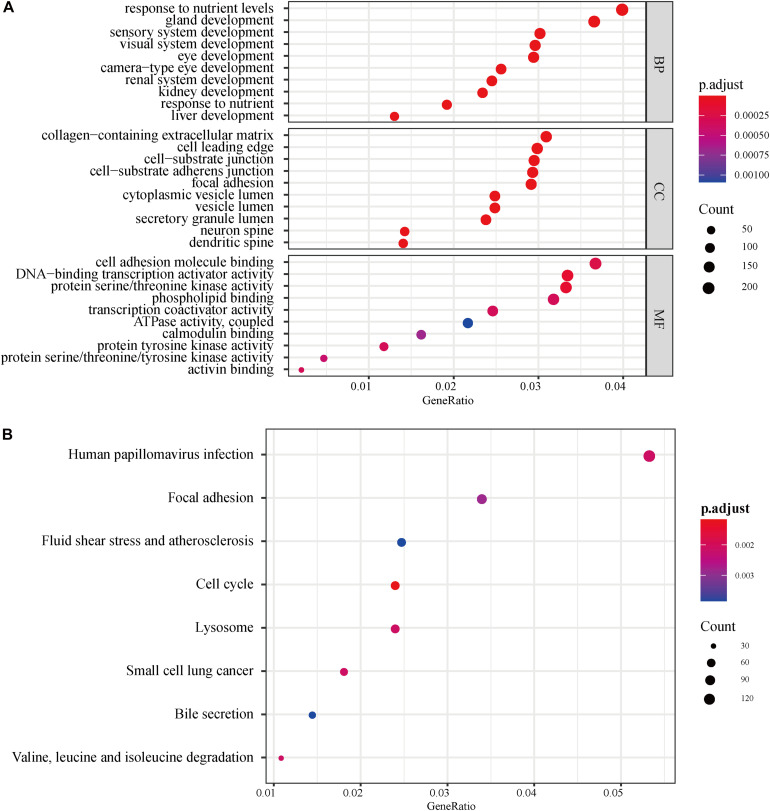
GO and KEGG pathway enrichment analyses of autophagy-related lncRNAs. **(A)** GO analysis results. The color of the dots represents the adjusted *P*-value: red, low; blue, high. The size of the dots represents the number of autophagy-related genes. **(B)** KEGG pathway enrichment analyses. The color of the dots represents the adjusted *P*-value, and the size of the dots represents the number of autophagy-related genes in the pathway. GO, gene ontology; KEGG, Kyoto Encyclopedia of Genes and Genomes; p.adjust, adjusted *P*-value; BP, biological process; CC, cellular component; MF, molecular function.

A total of 695 autophagy-related lncRNAs were identified by co-expression analyses. GO enrichment analyses of these autophagy-related lncRNAs revealed that significantly enriched biological processes included response to nutrient levels, gland development, sensory system development, and visual system development. Significantly enriched cell components included collagen-containing extracellular matrix, cell leading edge, and cell-substrate junction, while enriched molecular function terms included cell adhesion molecule binding, DNA-binding transcription activator activity, and protein serine/threonine kinase activity (Figure 2A). KEGG enrichment analyses uncovered an enrichment in pathways involved in responses to human papillomavirus infection, focal adhesion, fluid shear stress, and atherosclerosis (Figure 2B).

### Construction and Validation of the lncRNA-Based Prognostic Model

**FIGURE 3:**
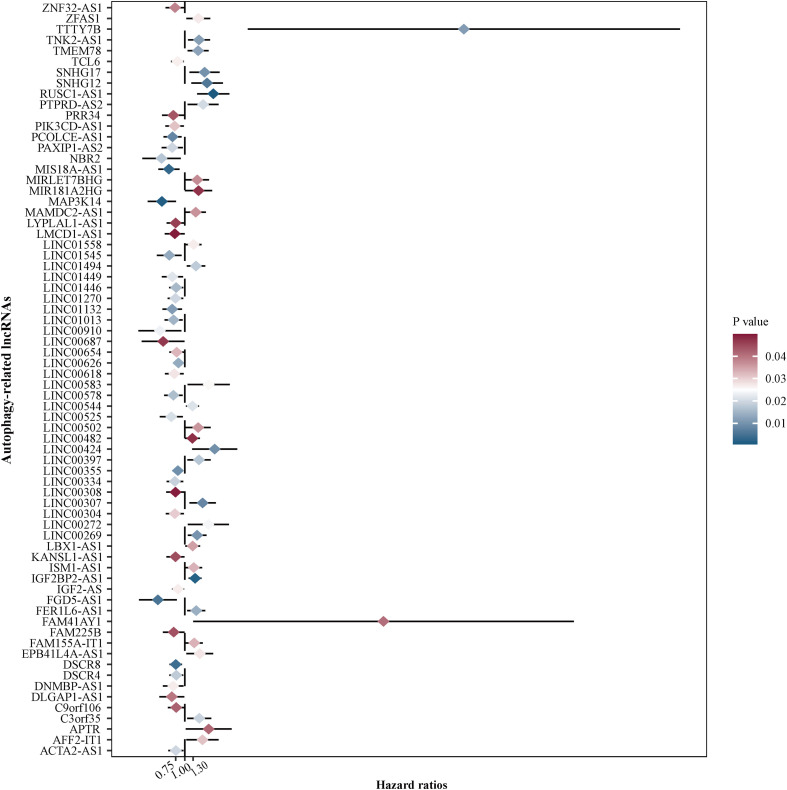
Identification of autophagy-related lncRNAs with prognostic value in osteosarcoma. We performed univariate Cox regression analyses and identified 69 autophagy-related lncRNAs associated with osteosarcoma patient prognosis. Red color represents high *P*-values, whereas blue color represents low *P*-values.

**FIGURE 4:**
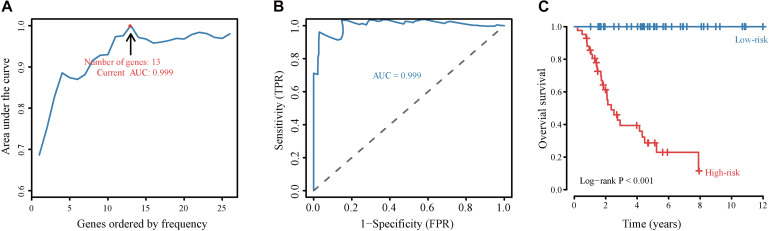
Construction and validation of the autophagy-related lncRNA signature-based prognostic model. **(A)** Identification of the autophagy-related lncRNA signature by iterative lasso Cox regression analyses. **(B)** ROC curve showing the prognostic performance of the autophagy-related lncRNA signature. **(C)** Kaplan-Meier survival analyses of patients in the high-risk score and the low-risk score groups. Patients with high scores (red line) have significantly worse overall survival than those with low scores (blue line); *P* < 0.05 was considered statistically significant.

**FIGURE 5:**
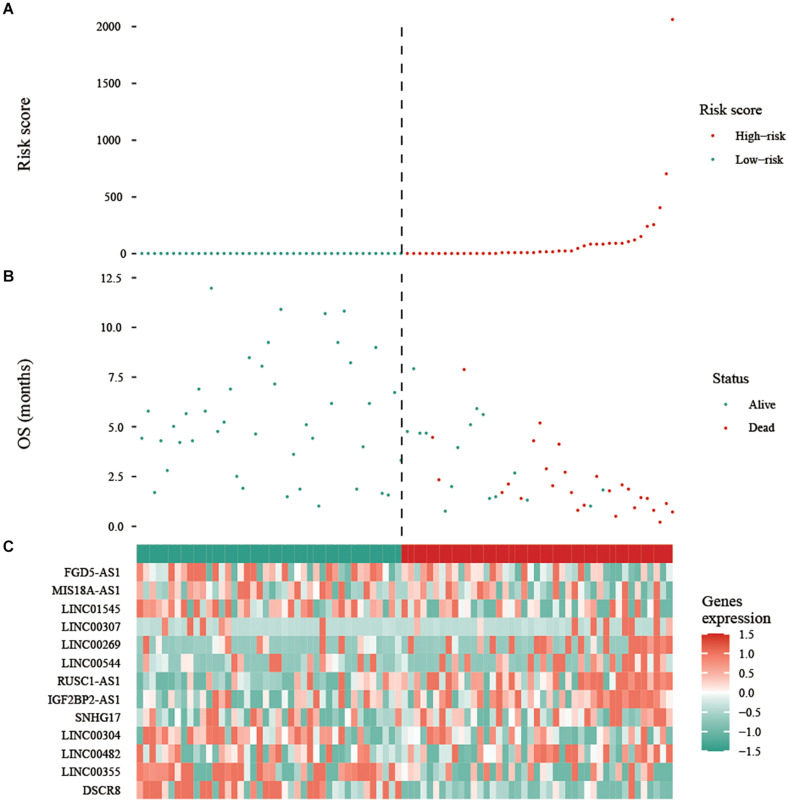
Expression of the prognostic autophagy-related lncRNA signature. **(A)** Risk score distribution of patients in the prognostic autophagy-related lncRNA signature. Red means high risk, green means low risk. **(B)** Survival status scatter plots for patients in the prognostic autophagy-related lncRNA signature. The green and red dots represent survival and death, respectively. **(C)** Expression patterns of risk genes in the prognostic autophagy-related lncRNA signature. Red means high expression, green means low expression. OS: overall survival.

To assess the prognostic value of autophagy-related lncRNAs in osteosarcoma, we performed univariate Cox regression analyses and identified 69 lncRNAs with significant prognostic value (Figure 3). To improve the accuracy of these results, we performed machine learning-based lasso Cox regression analyses using the 69 autophagy-related lncRNAs. We identified 13 lncRNAs (DSCR8, IGF2BP2-AS1, MIS18A-AS1, RUSC1-AS1, FGD5-AS1, LINC00269, LINC00307, LINC00355, LINC00482, LINC00544, LINC01545, SNHG17, and LINC00304) that were the most relevant to prognosis (Figure 4A and Table 2). ROC analyses yielded an AUC of 0.999, indicating the high prognostic value of these 13 lncRNAs (Figures 4A,B). Kaplan-Meier survival analyses revealed that the 5-year survival rate of patients in the high-risk group was significantly lower than that of patients in the low-risk group (*P* < 0.0001) (Figure 4C). Consistently, we found a significantly higher number of deaths in the high-risk group than in the low-risk group. LINC00482, SNHG17, IGF2BP2-AS1, RUSC1-AS1, LINC00544, LINC00269, and LINC00307 were expressed at higher levels in high-risk patients than in low-risk patients, whereas FDG5-AS1, MIS18A-AS1, LINC01545, LINC00304, LINC00355, and DSCR8 were expressed at lower levels (Figure 5).

**TABLE 2:** 13 autophagy-related lncRNAs identified as prognostic factors in osteosarcoma.

LncRNA symbol	Chromosome	Hazard ratio	z value	*P*-value
DSCR8	chr21:39,493,545–39,560,110	0.750810771	–2.875507459	0.004033786
IGF2BP2-AS1	chr3:185,430,316–185,447,575	1.387298726	3.106041191	0.001896103
MIS18A-AS1	chr21:32,277,863–32,284,702	0.603373955	–2.963909306	0.003037578
RUSC1-AS1	chr1:155,312,885–155,324,176	2.472771655	3.465331481	0.000529578
FGD5-AS1	chr3:14,877,562–14,949,563	0.425107546	–2.797958594	0.00514267
LINC00269	chrX:69,179,555–69,210,496	1.477790268	2.57481359	0.010029412
LINC00307	chr21:30,209,151–30,211,783	1.760759025	2.650425117	0.008039055
LINC00355	chr13:63,836,991–64,076,044	0.802730834	–2.597922769	0.009378959
LINC00482	chr17:81,299,220–81,309,250	1.271881871	1.978773849	0.047841474
LINC00544	chr13:29,935,771–29,950,488	1.278007987	2.303406918	0.021255959
LINC01545	chrX:46,886,154–46,912,377	0.610434289	–2.476977822	0.013250013
SNHG17	chr20:38,415,107–38,435,409	1.876304363	2.581594203	0.009834515
LINC00304	chr16:89,155,925–89,164,245	0.728438337	–2.165505394	0.030349003

### Autophagy-Related lncRNA Signature Validation and Comparison With Other Known Prognostic Biomarkers

**FIGURE 6:**
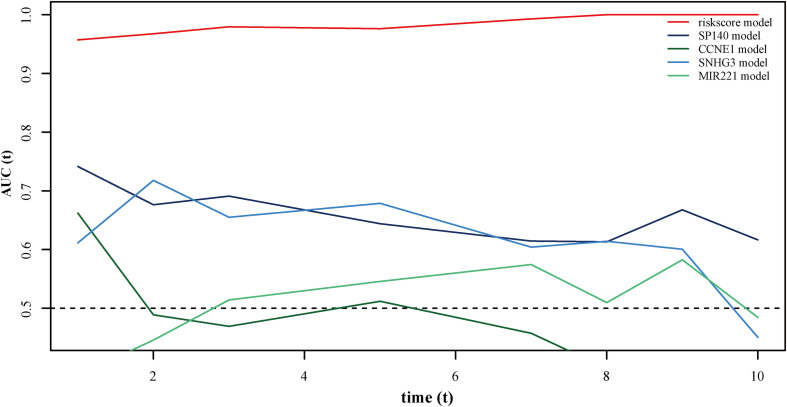
ROC curves showing the prognostic value of the autophagy-related lncRNA signature and other prognostic biomarkers in osteosarcoma patients.

Using data from the training cohort, we performed ROC analyses of the autophagy-related lncRNA signature along with other known prognostic biomarkers (SP140, CCNE1, SNHG3, and MIR221). Interestingly, the AUC of the autophagy-related lncRNA signature was higher than that of other known biomarkers (Figure 6), indicating that the autophagy-related lncRNAs signature is a better prognostic biomarker, providing a more accurate prediction of osteosarcoma patient survival.

### Assessment of the lncRNA Signature as an Independent Prognostic Factor and Nomogram Construction

**FIGURE 7:**
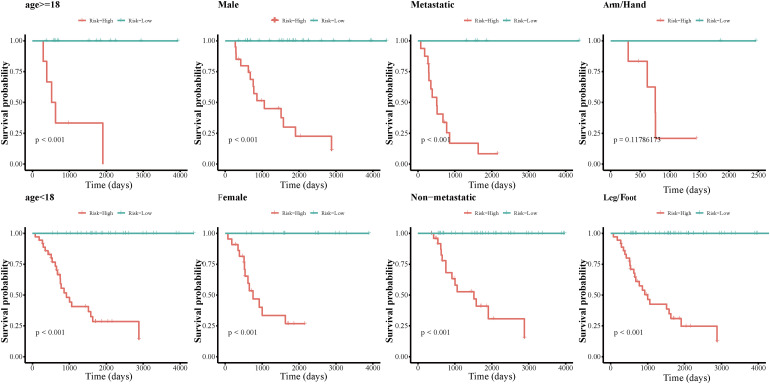
Kaplan-Meier survival analyses of the autophagy-related lncRNA signature in different osteosarcoma patient subgroups.

**FIGURE 8:**
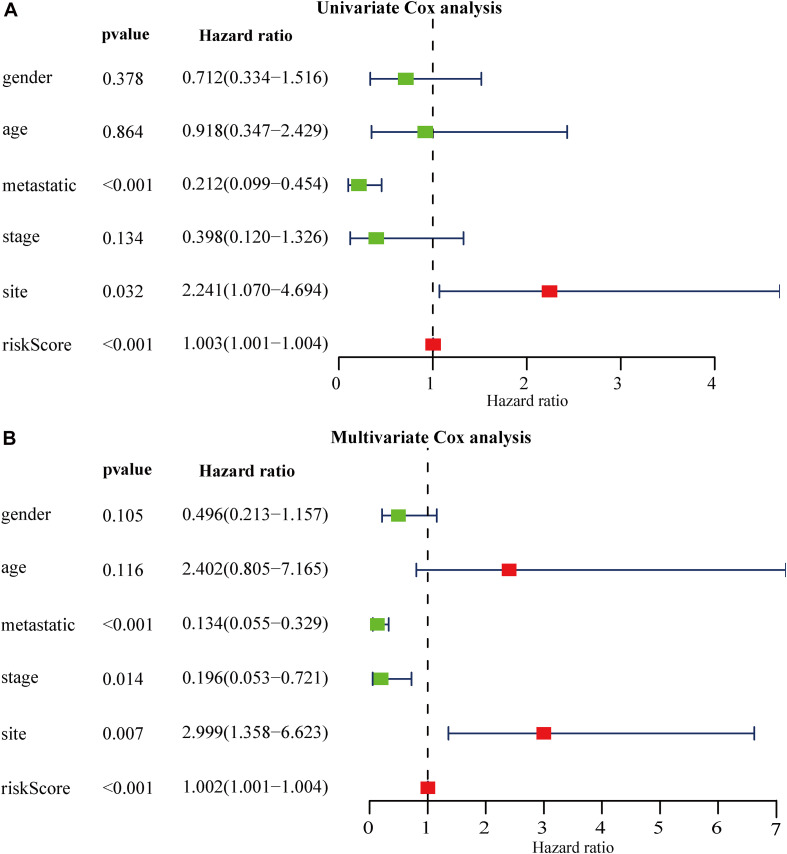
Relationship between autophagy-related lncRNA signature expression, clinicopathological characteristics, and osteosarcoma patient prognosis. **(A,B)** Forest plot showing the results of **(A)** univariate Cox regression analyses and **(B)** multivariate Cox regression analyses.

We grouped osteosarcoma patients based on different clinicopathological characteristics (age, sex, tumor location, and metastasis) and performed Kaplan-Meier survival analyses to assess the prognostic value of the autophagy-related lncRNA signature. We found that low-risk group patients lived significantly longer, regardless of age, sex, location, and metastasis (Figure 7). Univariate Cox regression analyses showed that tumor metastasis (hazard ratio [HR] = 0.212, *P* < 0.001), tumor location (HR = 2.241, *P* < 0.032), and expression levels of the 13 autophagy-related signature lncRNAs (HR = 1.003, *P* < 0.001) were significant predictors of osteosarcoma patient prognosis. Multivariate Cox regression analyses revealed that tumor metastasis (HR = 0.013, *P* < 0.001), stage (HR = 0.196, *P* = 0.014), tumor site (HR = 2.999, *P* = 0.007), and expression level of the autophagy-related lncRNA signature (HR = 1.002, *P* < 0.001) were significantly associated with osteosarcoma patient prognosis (Figure 8). These results suggest that the autophagy-related lncRNA signature is an independent prognostic factor in osteosarcoma patients. In addition, we combined the clinicopathological characteristics of the patients with the expression levels of the 13 autophagy-related lncRNAs to construct a nomogram (Supplementary Figure 1) and found that the signature provided an accurate prediction of osteosarcoma patient prognosis.

### GSEA and GSVA High- and Low-Risk Osteosarcoma Patients

**FIGURE 9:**
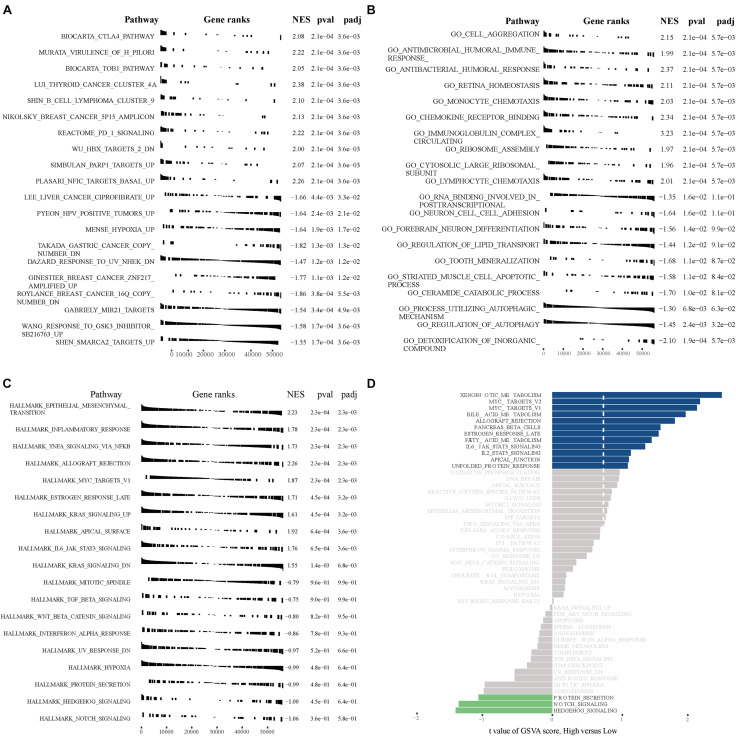
GSEA and GSVA in high- and low-risk osteosarcoma patients. **(A)** GSEA analyses with “c2.all.v7.1.symbols.gmt” as the reference gene set. **(B)** GSEA analyses with “c5.all.v7.1.symbols.gmt” as the reference gene set. **(C)** GSEA analyses with “h.all.v7.1.symbols.gmt” as the reference gene set. **(D)** GSVA analyses with “h.all.v7.1.symbols.gmt” as the reference gene set.

GSEA and GSVA were performed to identify important functional phenotypes between high- and low-risk osteosarcoma patients. GSEA revealed enrichment of gene sets associated with the CTLA4 pathway, TOB1 pathway, cell aggregation, antimicrobial humoral immune response, and EMT in high-risk osteosarcoma patients (Figures 9A–C). GSVA results indicated an enrichment of gene sets related to the MYC pathway, bile acid metabolism, allograft rejection, NOTCH signaling, and hedgehog signaling in high-risk osteosarcoma patients (Figure 9D).

### Analyses of Immune Cells in Osteosarcoma Tissues and Their Role in Osteosarcoma Patient Prognosis

**FIGURE 10:**
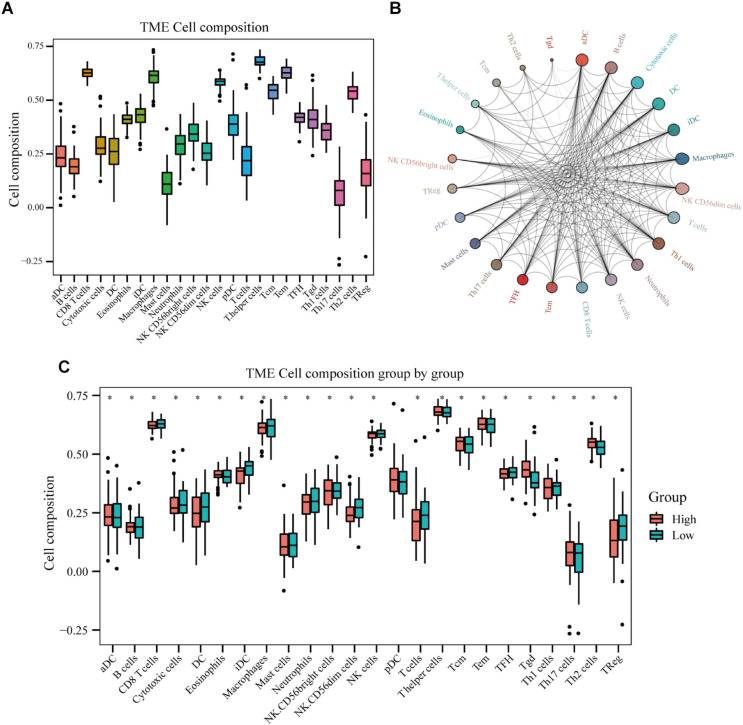
Immune cell infiltration in osteosarcoma tissues of high- and low-risk patients. **(A)** Results of immune cell infiltration composition analyses. **(B)** Immune cell interaction network. **(C)** Differences in immune cell infiltration between high- and low-risk osteosarcoma patients.

**FIGURE 11:**
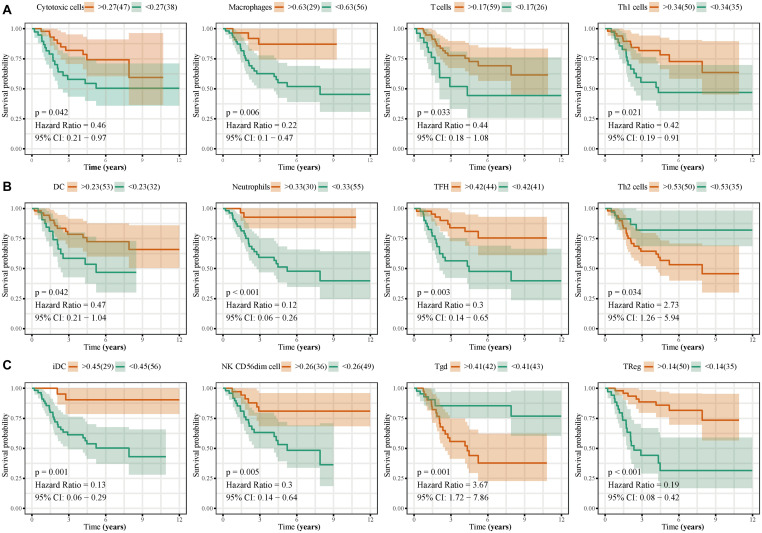
Effects of immune cell infiltration on the prognosis of osteosarcoma patients. Low levels of cytotoxic cells, macrophages, T cells, Th1 cells, DCs, neutrophils, TFH, iDC, NK CD56^*dim*^ cells, and Tregs, were associated with poor osteosarcoma patient prognosis. High levels of Th2 and Tgd cells were associated with poor prognosis in osteosarcoma patients.

Immune cell infiltration analyses revealed that T helper cells, effector memory T (T_*EM*_) cells, macrophages, CD8^+^ T cells, natural killer (NK) cells, and T helper 2 (Th2) cells were accumulated in osteosarcoma tissues, whereas the levels of infiltrating mast cells and regulatory T cells (Tregs) were low (Figure 10A). Immune cell interaction analyses indicated that dendritic cells (DCs), activated DCs (aDCs), immature DCs (iDCs), B cells, cytotoxic cells, and macrophages interacted strongly with other immune cells found in the tumor microenvironment (TME), in contrast to gamma delta T cells (Tgd), Th2 cells, central memory T (T_*C*__*M*_) cells, and T helper cells (Figure 10B). Interestingly, we found that the levels of eosinophils, NK cells, plasmacytoid DCs (pDCs), T_*CM*_, Tgd, and Th2 cells, were higher in the TME of high-risk group than of low-risk group. By contrast, cytotoxic cells, macrophages, T cells, Th1 cells, DCs, neutrophils, iDC, NK cells, and Tregs were found in lower levels in the high-risk group (Figure 10C). Next, we analyzed the effects of different immune cells on osteosarcoma patient prognosis and found that low levels of cytotoxic cells, macrophages, T cells, Th1 cells, DCs, neutrophils, iDCs, NK cells, follicular helper T (TFH) cells, and Tregs were associated with poor prognosis. High levels of Th2 and Tgd cells in the TME were associated with poor prognosis in osteosarcoma patients (Figure 11).

### Relationship Between Autophagy-Related lncRNAs and Immune Cell Infiltration

**FIGURE 12:**
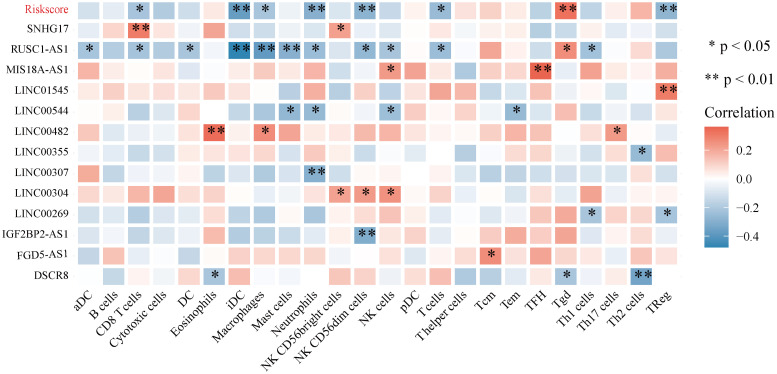
Correlation between the 13 autophagy-related lncRNAs and immune cell infiltration.

Correlation analyses between autophagy-related lncRNAs and infiltrating immune cells revealed that the expression levels of SNHG17, LINC00482, MIS18A-AS1, and LINC01545 were positively correlated with the levels of CD8^+^ T cells, eosinophils, TFH cells, and Tregs, respectively. RUSC1-AS1 expression levels were negatively correlated with the levels of infiltrating iDCs, macrophages, and mast cells. In addition, LINC00307, IGF2BP2-AS1, and DSCR8 levels were negatively correlated with the levels of neutrophils, NK CD56^*dim*^ cells, and Th2 cells, respectively. Furthermore, the expression levels of the autophagy-related lncRNA signature were positively correlated with Tgd levels and negatively correlated with NK CD56^*dim*^, neutrophil, Treg, and iDC levels (Figure 12).

## Discussion

Osteosarcoma is a common bone malignancy in young adults and children. It is characterized by high rates of early distant metastasis, high mortality rate, and extremely poor prognosis. Osteosarcoma patients often require aggressive treatments and frequent monitoring. Despite the need for robust biomarkers, most candidate biomarkers exhibit unsatisfactory prognostic and predictive performance. The most well-established prognostic markers used in a clinical setting are predominantly based on tumor features, such as AJCC-TNM classification. In osteosarcoma, however, the sensitivity, specificity, and accuracy of these factors are limited. Recent studies have shown that aberrant expression of lncRNAs is associated with osteosarcoma development, progression, and metastasis, and thus can be used as a predictive and prognostic biomarker in osteosarcoma (Lu et al., 2019; Negri et al., 2019). In this study, for the first time, we analyzed the sequencing data of osteosarcoma samples from the TARGET database using various bioinformatic tools and machine learning algorithms to establish a robust autophagy-related lncRNA signature to predict prognosis in osteosarcoma patients.

We initially identified a total of 695 autophagy-related lncRNAs. We found that these were enriched in functional terms related to response to nutrient levels, gland development, and sensory system development, cell adhesion, and protein serine/threonine kinase activity. Cell leading edge (Perike et al., 2014), focal adhesion (Gu and Zhou, 2018), cell adhesion molecule binding (Du et al., 2018), protein serine/threonine kinase activity (Pruksakorn et al., 2018), phospholipid-binding (Jiang et al., 2018), ATPase activity (Meszaros and Karin, 1995), and protein tyrosine kinase activity (Kostas et al., 2018) have been previously shown to be associated with osteosarcoma development and metastasis. Consistent with the GO enrichment findings, enriched KEGG pathways included focal adhesion and lysosomal degradation. Studies have shown that certain focal adhesion molecules promoted tumor progression and were associated with poor clinical outcomes in osteosarcoma patients (Gu and Zhou, 2018).

Using univariate Cox and iterative lasso Cox regression analyses we identified 13 lncRNAs (DSCR8, IGF2BP2-AS1, MIS18A-AS1, RUSC1-AS1, FGD5-AS1, LINC00269, LINC00307, LINC00355, LINC00482, LINC00544, LINC01545, SNHG17) with prognostic value in osteosarcoma. Interestingly, abnormal expression of DSCR8 is associated with poor prognosis in the liver (Wang et al., 2018) and ovarian cancer (You et al., 2020). Abnormal expression of RUSC1-AS1 has also been implicated in the development of cervical cancer (Guo et al., 2020). FGD5-AS1 has been shown to promote colorectal cancer cell proliferation, migration, and invasion by functioning as a sponge for miR-302e and regulating CDCA7 expression (Li et al., 2019). Similarly, LINC00355 has been shown to act as an miR-195 sponge, thereby upregulating HOXA10 and promoting migration, invasion, and EMT in head and neck squamous cell carcinoma (Lu et al., 2020). Importantly, we compared the prognostic ability of these 13 autophagy-related lncRNAs with those of other known prognostic biomarkers in an independent cohort (using the GSE39055 dataset). The autophagy-related lncRNA signature predicted osteosarcoma patient prognosis with high sensitivity and specificity and independently of age, sex, tumor location, and tumor metastasis. Consistently, univariate and multivariate Cox regression analyses indicated that the autophagy-related lncRNA signature was an independent prognostic factor in osteosarcoma patients. We also integrated the signature and clinicopathological factors to construct a nomogram and confirmed that our autophagy-related lncRNA signature was an independent and robust prognostic biomarker and could be used to monitor osteosarcoma patients.

The CTLA4, TOB1, MYC, Notch, Hedgehog, and EMT pathways play a critical role in cancer progression (Artym et al., 2003; Hamester et al., 2019; Jiang K. et al., 2019; Karacosta et al., 2019; Kalbasi and Ribas, 2020). Intriguingly, GSEA/GSVA results indicated that the 13 autophagy-related lncRNAs were predicted to affect all of these pathways. Wang et al. (2016) reported that CTLA-4 is constitutively expressed in memory T cells and Tregs promoting osteosarcoma progression. MYC-driven signals have also been linked to osteosarcoma development, and targeting the MYC-driven super-enhancer signaling has emerged as a promising treatment strategy for osteosarcoma patients (Chen D. et al., 2018). Canalis (2018) showed that changes in Notch signaling are associated with osteosarcoma development, as well as with the metastatic potential of breast and prostate cancer cells. Zhao Z. et al. (2018) showed that the aberrant activation of the Hedgehog pathway is also associated with enhanced osteosarcoma growth and metastasis. In accordance with these previous findings, we found that the autophagy-related lncRNA signature was significantly associated with osteosarcoma initiation and progression.

It has become widely accepted that immune cell infiltration in the TME regulates various tumor features, including tumor malignancy and metastatic potential (Manandhar and Lee, 2018; Steven and Seliger, 2018; Lee and Cho, 2020). Here, we analyzed the composition of immune cells in the osteosarcoma TME. We found elevated levels of macrophages, CD8^+^ T cells, NK cells, and Th2 cells in osteosarcoma tissues, whereas the levels of mast cells and Tregs were very low. Interestingly, high-risk group patients exhibited higher levels of eosinophils, NK cells, pDCs, T_*CM*_, Tgd, and Th2 cells compared to low-risk group patients. On the other hand, the levels of infiltrating cytotoxic cells, macrophages, T cells, Th1 cells, DCs, neutrophils, TFH cells, iDCs, NK CD56^*dim*^ cells, and Tregs were lower in patients that expressed high levels of the autophagy-related lncRNA signature than those that expressed low levels of it. We also found that low levels of infiltrating cytotoxic cells, macrophages, T cells, Th1 cells, DCs, neutrophils, TFH cells, iDCs, NK CD56^*dim*^ cells, and Tregs were associated with poor prognosis in osteosarcoma patients. High levels of infiltrating Th2 and Tgd cells were also associated with poor outcomes. Macrophages have been shown to accumulate in osteosarcoma tissues, establishing an immunotolerant TME that facilitates tumor progression (Heymann et al., 2019). During osteosarcoma progression, macrophages in the TME are reprogrammed toward an M2-like phenotype, further promoting osteosarcoma growth (Kelleher and O’Sullivan, 2017). Clinical studies have shown that in osteosarcoma patients, the number of circulating NK cells in the peripheral blood is significantly reduced, suggesting that NK cells play an important role in the development of osteosarcoma (Tullius et al., 2020). In addition, ANGPTL2 secretion by tumor cells has been demonstrated to recruit neutrophils into the lungs, triggering osteosarcoma lung metastasis (Charan et al., 2020). In this study, we analyzed the relationship between the 13 autophagy-related lncRNAs and immune cell infiltration and found that the expression levels of SNHG17, LINC00482, MIS18A-AS1, and LINC01545 were positively correlated with the levels of CD8^+^ T cells, eosinophils, TFH cells, and Tregs, respectively. RUSC1-AS1 levels were negatively correlated with the levels of infiltrating iDCs, macrophages, and mast cells. Similarly, LINC00307, IGF2BP2-AS1, and DSCR8 expression levels were negatively correlated with the levels of infiltrating neutrophils, NK CD56^*dim*^ cells, and Th2 cells, respectively. Most importantly, we found that the expression levels of the autophagy-related lncRNA signature were positively correlated with the levels of infiltrating Tgd cells and negatively correlated with the infiltration levels of NK CD56^*dim*^ cells, neutrophils, and iDCs. However, the mechanisms underlying the effects of autophagy-related lncRNAs and immune cell infiltration remain poorly understood and merit further investigation.

Despite the novelty and significant clinical implications of our findings, our study had several limitations. First, we only analyzed data from the TARGET database, and the sample size was relatively small. Second, although we provide some evidence regarding the possible mechanisms by which autophagy-related lncRNAs affect prognosis, further comprehensive studies are required to elucidate the precise mechanisms underlying the prognostic effects of these autophagy-related lncRNAs in osteosarcoma. Finally, our results are based on bioinformatic analyses and require experimental validation in large-cohort clinical studies.

## Conclusion

We performed comprehensive bioinformatics analyses of expression data from osteosarcoma patients and developed a novel signature composed of 13 autophagy-related lncRNAs. Compared to clinically used prognostic biomarkers, the autophagy-related lncRNA signature provides a more robust and accurate prediction of osteosarcoma patient prognosis. We also show that the expression of these 13 autophagy-related lncRNAs may promote osteosarcoma development and progression by regulating immune cell infiltration in the TME. Therefore, this autophagy-related lncRNA signature may represent a promising prognostic biomarker in osteosarcoma.

## Data Availability Statement

The original contributions presented in the study are included in the article/Supplementary Material, further inquiries can be directed to the corresponding author/s.

## Author Contributions

G-ZZ, Z-LW, E-HR, and Q-QX conceived and designed the study. C-YL, W-HY, and Y-JD contributed to dataset selection and bioinformatics analysis. G-ZZ and Q-QX wrote and revised the manuscript. All authors read and approved the final manuscript.

## Conflict of Interest

The authors declare that the research was conducted in the absence of any commercial or financial relationships that could be construed as a potential conflict of interest.

## Footnotes

1 https://ocg.cancer.gov/programs/target

2 http://autophagy.lu/clustering/index.html

3 https://CRAN.R-project.org/package=glmnet

## Supplementary Material

Supplementary Figure 1 Prognostic nomogram for OS patients.

## Abbreviations

OS: osteosarcoma
TARGET: Therapeutically Applied Research To Generate Effective Treatments
GEO: gene expression omnibus
GO: gene ontology
KEGG: Kyoto encyclopedia of genes and genomes
PPI: protein-protein interaction
HADb: human autophagy database
BP: biological process
CC: cell components
MF: molecular function
HR: hazard ratio
Lasso: least absolute shrinkage and selection operator.
