# Supplementary figures and images for: Development of a Machine Learning-Based Autophagy-Related lncRNA Signature to Improve Prognosis Prediction in Osteosarcoma Patients
Source: Front Mol Biosci. 2021 May 21;8:615084. doi: 10.3389/fmolb.2021.615084 (PMC8176230; doi:10.3389/fmolb.2021.615084)

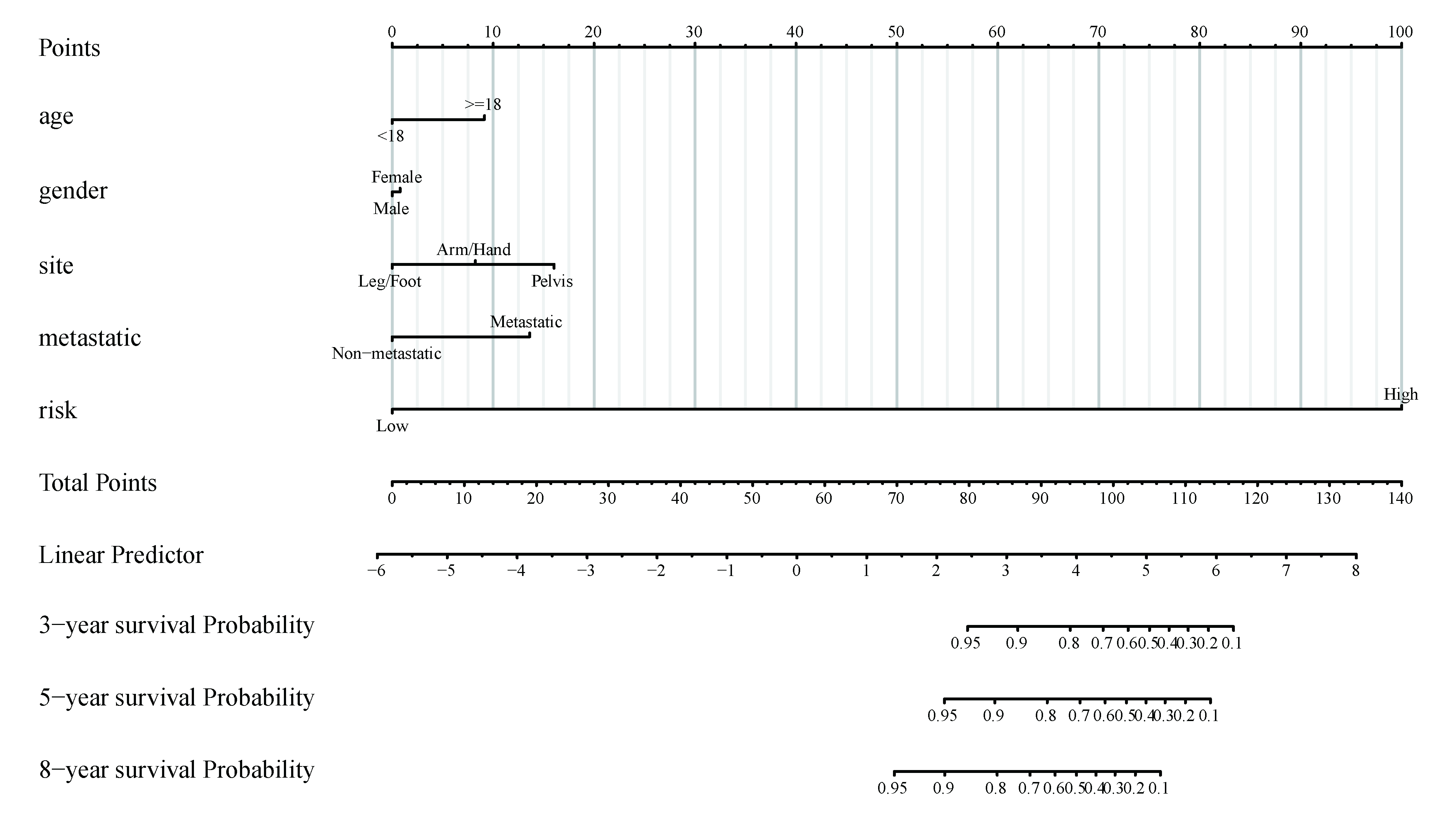

Supplement: Supplementary Figure 1 — Prognostic nomogram for OS patients. [file Image_1.tif]
